# Squamous cell carcinoma of the nasal vestibule: a diagnostic and therapeutic challenge

**DOI:** 10.1007/s00405-024-08813-8

**Published:** 2024-07-23

**Authors:** Gabriele Testa, D. Mattavelli, V. Rampinelli, C. Conti, C. Piazza

**Affiliations:** 1 Unit of Otorhinolaryngology – Head and Neck Surgery, ASST Spedali Civili di Hospital, University of Brescia, Piazza Spedali Civili 1, Brescia, 25123 Italy; 2 Department of Medical and Surgical Specialties, Radiological Sciences, and Public Health, University of Brescia, Brescia, Italy

**Keywords:** Nasal vestibule carcinoma, Head and neck cancer, Squamous cell carcinoma, Brachytherapy, Interventional radiotherapy, Rhinectomy, Review

## Abstract

Nasal vestibule squamous cell carcinoma (NVSCC) is an exceedingly rare malignancy, often misclassified due to its anatomical location and lack of a standardized definition. This review aims to consolidate current evidence on NVSCC, focusing on epidemiology, risk factors, classification, clinical presentation, treatment modalities, and prognostic factors. The NV anatomy is delineated, emphasizing the need for a clear definition to avoid misclassification. Risk factors include smoking, sunlight exposure, and debated associations with chalk exposure or viral factors. Clinical presentation includes symptoms like nasal obstruction, pain, burning, and bleeding, often misdiagnosed as inflammatory conditions. NVSCC exhibits distinct local spread patterns along cartilaginous surfaces, with the facial and submandibular lymph nodes at higher metastatic risk. Current classifications lack consensus, hindering comparison of outcomes. Treatment varies, with surgery or radiotherapy for early-stage tumors and multimodality approaches for advanced cases. The choice between surgery and radiotherapy is debated, with potential advantages and drawbacks for each. Radiotherapy, especially with Interventional RadioTherapy (IRT, previously known as brachytherapy), is gaining prominence, showing promising outcomes in terms of local control and cosmetic results. Prophylactic neck treatment remains controversial, with indications based on tumor characteristics. Prognostic factors include T classification, tumor size, surgical margins, nodal involvement, and histological features. Long-term survival rates range widely, emphasizing the need for further studies to refine management strategies for this rare malignancy. In conclusion, NVSCC poses diagnostic and therapeutic challenges, warranting multidisciplinary approaches and continued research efforts to optimize patient outcomes.

## Introduction

The nasal vestibule (NV) forms most anterior part of the nasal cavity and can be defined as a pear-shaped space bounded laterally by the alar cartilage, anteriorly by the muco-cutaneous junction and posteriorly by the limen nasi [1, 2]. The medial part of the nasal vestibule includes the columella, the medial crura of alar cartilage and the septum.

Malignancies arising from this district are extremely rare, and squamous cell carcinoma (SCC) is the most common entity [3].

The anatomical location and the rarity of the disease contributes to further underestimate their incidence, as these malignancies are often misclassified either as skin or sinonasal cancers (i.e., arising from the septum) [4]. Furthermore, disagreement on the staging system to apply makes also difficult to compare published data [5].

**Fig. 1:**
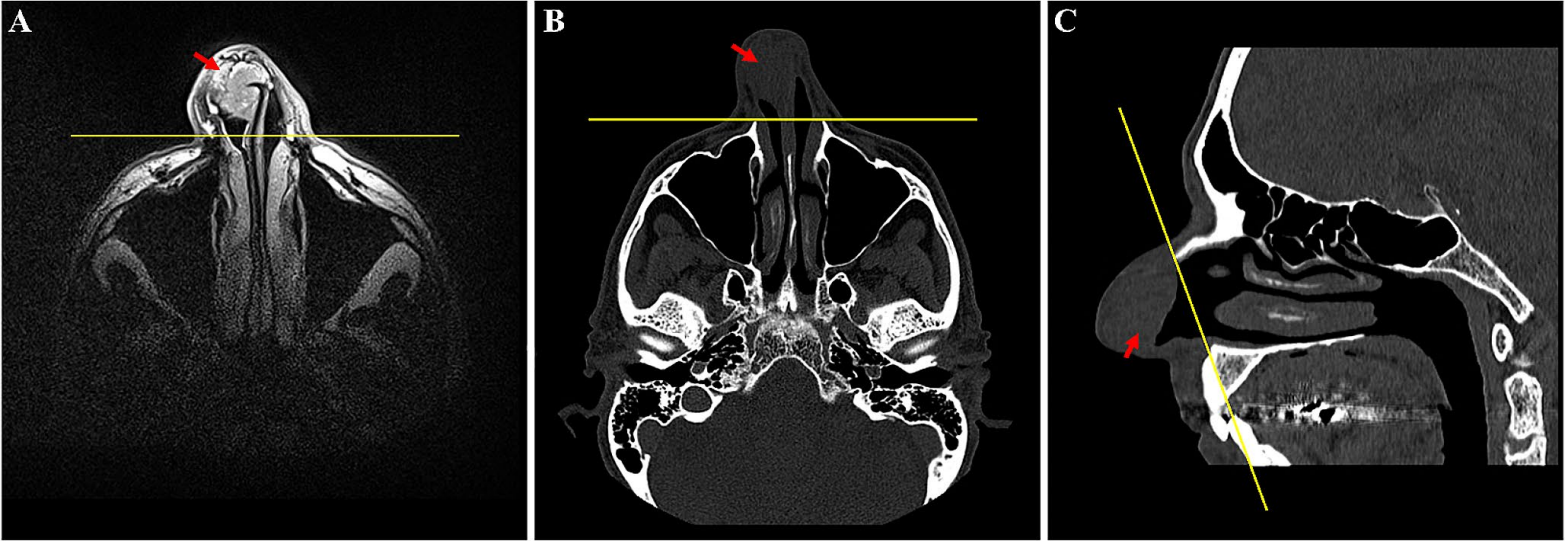
Pretreatment imaging of a patient diagnosed with a right NVSCC, limited to the NV, anterior to a line passing through the piriform aperture, staged as T1 according to Wang and T2b according to Bussu et al. [21]. Axial MRI T2-weighted sequence (**A**) shows septal cartilage infiltration of the tumor. CT-scan images (axial, **B** and sagittal, **C**) allow clear identification of the bony piriform aperture

When dealing with SCC of the nasal vestibule (NVSCC), it is paramount to clearly define the site of origin of the tumour to avoid any misclassification with skin or nasoethmoidal cancers. Therefore, a conventional and easy-to-apply definition of the boundaries of the nasal vestibule is eagerly warranted. To meet this need, Bussu et al. recently proposed a simplified but reproducible demarcation of the NV [6] by the identification of three limits: a plane tangential to the piriform opening as the posterior boundary, easily identifiable also through imaging studies (Fig. 1) and demarcating NV from proper nasal cavity, the limen nasi, separating it from the skin of the nose, and the edge of the nostril, dividing it from the upper lip.

The aim of the present paper is to evaluate the current evidence on this topic, with a particular focus on epidemiology, aetiology, classification, treatment options and oncological outcomes. A comprehensive review of studies published since 2000 via via PubMed (www.pubmed.gov; National Center for Biotechnology Information, National Library of Medicine, USA) on the topic was conducted (Table 1).

**Table 1:** Literature overview regarding SCC of NV

Author	Year	*N* of patients	T classification	Treatment on T	Treatment on *N*	Reconstruction	Regional metastases	Survival rates	Local control	Regional control	Conclusion
**Filtenborg** [7]	2022	162	UICC	Surgery, RT, Surgery + RT.	No	-	At diagnosis: 3,7% During FU: 38%	5-y OS: 60%	5-y Local-regional failure: 28% (64% on T, 38% on N) 5-y ultimate LRF: 13%		- Stage was a significant independent prognostic factor. - No difference in LRF, DSM or OS were shown between curative treatments. - Failure most common at T.
**Scheurleer** [8]	2022	68	Wang, UICC	RT (BT or BT + EBRT)	Dissection or dissection + RT (2 pt cN+)	-	At diagnosis: 3%	5-y OS: 66,2%	5-y Local-regional control: 91,1% (All recurrence within 3 years)		- BT offers excellent oncological outcomes treatment for early-stage T. - Recurrences occur within 3 years after treatment. - T stage (wang) and T diameter (15 mm) have a significant correlation with 5-y LRFS.
**Eberle** [9]	2022	17 with SCC (of 21)	Wang, UICC	RT (Carbon ion beam RT combined with VMAT, CIRT-B) alone or with CHT (platinum-based)	For advanced stage and cN+	-	At diagnosis: 14.3% (included + 4 patients with other histology)	2-y organ-preservered survival: 83,3% (included + 4 patients with other histology)	2-y local control: 84% (included + 4 patients with other histology)	-	CIRT-B + VMAT in malignant tumors of the nasal vestibules is a good option as organ preserving therapy.
**Chabrillac** [10]	2022	23 (SCC involving NV)	Wang, AJCC	Surgery (TR) alone or with adjuvant RT or CRT	Dissection (cN0, advanced T). Dissection or dissection + RT (cN+).	Prosthesis (*n* = 21, 91% of pt)	During FU: 13%	5-y OS: 67,5%	-	-	- Positive excision margins are predictive factor for tumour recurrence. - SCCs involving the nasal vestibule that are not amenable to limited surgical resection, TR along with radiotherapy provide good oncological outcomes and should be considered the main treatment option
**Czerwinski** [11]	2021	225	Wang	RT (BT and EBRT)	Dissection and RT or RT (4 pt cN+)	-	At diagnosis: 1,8% During FU: 9,8%	3-y OS: 82%	3-y local control: 87% 3-y ultimate LC: 96%	3-y regional control: 83% 3-y ultimate RC: 95%	- T > 1.5 cm and T2 stage significant risk factors for regional recurrence. - BT achieved superior LC and SPN (Survival with Preserved Nose) compared with EBRT for T1-T2.
**Lambertoni** [12]	2021	45	Wang, AJCC	Surgery, Surgery + RT.	Dissection or RT (4 pt cN0)	Local flap (*n* = 25), regional flap (*n* = 11), free flap (*n* = 6), graft (*n* = 8), Prosthesis (*n* = 2), none (*n* = 5), others (*n* = 8)	During FU: 8,8%	5-y OS: 81,9%	5-y Local Control: 72,4%	5-y Regional Control: 92,1%	- Wang’s classification, site of origin, extent of surgery and margins status significantly correlated with prognosis and recurrence rate. - Adj-RT and elective neck treatment suggested for high risk patients (Wang T2-T3, G2-G3)
**Tagliaferri** [13]	2020	14	Wang	BT	Neck dissection (2 pt cN+)	-	At diagnosis: 14,3%	3-y OS: 69,2%	3-y Local Control: 85,7%	3-y Regional Control: 81,2%	- Wang stage was statistically significant on local control (LC), disease-free survival (DFS), disease-specific survival (DSS), and overall survival (OS). - BT could be considered as a definitive treatment in nasal vestibule cancer with excellent oncological and cosmetic outcomes.
**Bussu** [4]	2020	19	Wang	BT	Dissection (1 pt cN+)	-	At diagnosis: 5,3%	5-y DSS: 92,3%	-	-	- BT is effective for primary cT1-2 NVSCC. - Nasal function and cytological findings are better in patients treated by BT than in those treated by EBRT.
**Czerwinski** [14]	2018	102	Wang	BT	Dissection and RT or RT (2 pt cN+)	-	At diagnosis: 1,9%	5-y OS: 72% 5-y DSS: 94%	5-y Local Control: 95% 5-y ultimate LC: 98%	5-y Regional Control: 91% 5-y ultimate RC: 96%	- BT is recommended for cT1-2 NVSCC (excellent local and regional tumor control, high patient satisfaction). - Tumor volume (> 2,3cm^3^) is a prognostic factor for regional recurrence.
**Vital** [15] [16]	2017/ 2018	46	UICC	Surgery, Surgery + RT, RT.	-	-	-	5-y OS: 73,1% 5-y DSS: 92%	-	-	Prognostic factors for NVSCC: - Aberrant p53 pattern (worse outcome). - Positivity for PD-L1 in tumor cells (better outcome).
**Zaoui** [17]	2017	26	Wang, UICC	Surgery, Surgery + RT.	Dissection (9 cN0)	Prosthesis (*n* = 15), regional flap (*n* = 5) local flap (*n* = 7), other (*n* = 2)	At diagnosis: 3,8% During FU: 7,6%	5-y OS: 96,2% 5-y DSS: 96,2%	Local recurrence: 4%	Regional recurrence: 8%	Primary open surgery is a valuable treatment option for NVSCC, all stages.
**Vanneste** [18]	2016	81	Wang	RT	-	-	During FU: 10%	5-y OS: 59%	5-year local control: T1 97%, T2 68%, T3 53%	-	- Wang stage significant factor on local control. - Local radiotherapy for T1, EBRT for T2/3, better cosmetic results.
**Bussu** [19]	2016	12	Wang	Surgery, BT.	Dissection (2 cN0)	Epithesis (*n* = 2), local flaps (*n* = 3)	At diagnosis: 0% During FU: 17%	2-y OS: 83% for surgery, 80% for BT	-	-	Surgery and BT have comparable oncological results, BT has better aesthetic results.
**Wray** [20]	2016	99	AJCC	RT (EBRT, BT, EBRT + BT or Surgery + EBRT)	Dissection or RT (cN + + 12 cN0)	-	At diagnosis: 5% During FU: 11%	5-y OS: 76% 10-y OS: 53%	5-y Local-ragional control: 77% 5-y local control: 87% 5-y regional control: 87%		- T1-T2: RT provides good cosmetic and curative results; surgery is a reasonable alternative if adequate cosmetic and functional results can be achieved. - T4: Surgery + RT involving bone Elective neck treatment recommended only for T4
**Lipman** [21]	2015	60	Wang	RT	No	-	At diagnosis: 0% During FU: 7%	5-y OS: 60%	3-y Local control: 91%	3-y Regional control: 93%	- Wang stage significant factor on local control. - BT for T1-T2 with accettable toxicity and aesthetics; in case of recurrence, surgery is indicated.
**Koopmann** [22]	2015	30	Wang, UICC	Surgery, Surgery + RT.	Dissection (22 cN+)	-	At diagnosis: 0% During FU: 3%	5-y OS: 92% 5-y DFS: 91,7%	-	-	- Elective treatment on N not recommended for early stage. - Grading is a prognostic factor for OS (worse in T3).
**Vital** [23]	2015	30	UICC	Surgery, Surgery + RT, RT.	Dissection, RT or comb.	Local flap (*n* = 2), composite graft (*n* = 4), paramedian forhead flap (*n* = 3), none (*n* = 5)	At diagnosis: 7% During FU: 7%	-	5-y Local-regional control: 70%		- Advanced T4 carcinomas show a high recurrence rate. - Positive surgical margins are the main predictor for a locoregional recurrence.
**Ledderose** [24]	2014	10	Wang	Surgery, Surgery + RT.	Dissection (2 cN+)	No reconstruction	At diagnosis: 10% During FU: 0%	-	No recurrence detected (mean FU 37,6 months)		Endoscopic resection for selected T1-T2 achieve good results
**Agger** [25]	2009	174	UICC/AJCC, Wang	Surgery, RT, Surgery + RT.	Dissection or RT or comb.(cN+)	Free flaps, local flaps, epithesis	At diagnosis: 6% During FU: 7%	5-y OS: 50% 5-y DSS: 74%	5-y Local-regional control: 67%		- Wang class. is more prognostic and easier to use than the UICC. - Surgery or hypofractionated RT can be used for T1 lesions, whereas larger lesions should be treated with combined approach. - T and N significant prognostic factors for locoregional control.
**Downley** [26]	2008	27	UICC, Wang	Surgery, RT, Surgery + RT.	Dissection, RT or comb. (cN+)	-	During FU: 15%	5-y OS: 68%	5-y Local-regional control: 58%		Surgery or surgery + RT lead to better outcomes for T2-T3 Wang) than exclusive RT
**Barasan** [27]	2007	10	AJCC	Surgery	Dissection (3 cN+, 4 cN0)	Paramedian forhead flap (*n* = 3), nasolabial flap (*n* = 2), aural composite graft (*n* = 1), split thickness skin flap (*n* = 1)	At diagnosis: 33%	3-y OS: 66%	3-y Local-regional control: 80%		Surgery is a successful therapeutic modality
**Wallace** [28]	2007	79	AJCC	RT, Surgery + RT.	Dissection, RT or comb. (cN+)	-	At diagnosis: 7% During FU: 13%	5-y OS: 76%	5-y Local-regional control: 77% 5-y local control: 87%		- RT has high cure rate for T1–T2 and favorable T4 tumors. - Surgery and RT result in an extensive T4.
**Jeannon** [1]	2007	84	UICC, Wang	Surgery, RT, Surgery + RT.	Dissection, RT or comb.	Nasolabial flap (*n* = 4), forehead flap (*n* = 8), free skin graft (*n* = 5), local flaps (*n* = 2), composite graft (*n* = 4), none (*n* = 3).	At diagnosis: 8%	5-y OS: 58%	-	-	- T and N-stage are significantly associated with OS. - Wang classification is more significant.
**Levendag** [29]	2006	64	Wang	BT	No	-	At diagnosis: 0% During FU: 0%	5-y OS: 59%	5-y Local Control: 92%	-	RT for T1-2, surgery for T3
**Langendijk** [30]	2004	56	Wang	RT (EBRT, EBRT + BT)	No	-	At diagnosis: 0% During FU: 12%	5-y OS: 66%	2-y Local control: 79% 5-y ultimate LC: 95%	2-y regional control: 87% 5-y ultimate RC: 97%	- RT adeguate for T1-2 - No elective neck therapy in cN0
**Kummer** [31]	2002	47	Wang	RT (EBRT, EBRT + BT, BT)	-	-	During FU: 13%	-	5-y LC for T1-T2: 91% 1-Y LC for T3: 0%	2 with regional ultimate failure	- Wang stage significant factor on local control. - Effect of RT correlated with T-stage (less success in T3).
**Fornelli** [32]	2000	32	No classification applied	Surgery, RT, Surgery + RT.	RT (6 cNo)	-	At diagnosis: 0% During FU: 41%	5-y OS: 50%	5 local recurrence detected	13 regional recurrence detected	Combined treatment (surgery + RT) and elective irradiation of facial and cervical lymph nodes is advocated for advenced tumors
**Samaha** [33]	2000	14	No classification applied	Surgery, RT, Surgery + RT.	-	Flap (*n* = 3)	At diagnosis: 7% During FU: 21%	2-y OS: 57%	2-y Local-regional control: 42%		- Local and regional recurrence are poor prognostic factors - Delay flap reconstruction for a minimum of 12 months

UICC, Union International Cancer Control; AJCC, American Joint Committee on Cancer; RT, Radiotherapy; EBRT, External Beam RT; FU, Follow-Up; OS, overall survival; LRF, Local-Regional failure; DSM, Disease-Specific Mortality; BT, Brachytherapy; EBRT, External Beam Radiotherapy; LRFS, Locoregional recurrence-free survival; VMAT, Volumetric Modulated Arc Therapy; CHT, Chemotherapy; SCC, squamous cell carcinoma; NV, nasal vestibule; TR, total rhinectomy; LC, Local Control; RC, Regional Control; DSS, Disease-Specific Survival; PD-L1, programmed death-ligand 1

### Incidence and clinical presentation

NVSCC accounts for less than 1% of all head and neck cancers [3]. The estimated incidence is 2–4 per million inhabitants and is based on retrospective studies from Denmark and England (0,32 per 100 000 habitants for DAHANCA group, 1,99 per million population for Dowley et al.) [25, 26]. According to the larger studies, the peak of incidence in the 7th decade, with a mean age ranging from 67 to 71 years [7, 11, 25]. Male-to-female ratio is variable, but in general there is a slightly higher incidence in men (M: F ratio of ~ 1,1–1,2:1) [7, 8, 25]. Patients can present with symptoms of nasal obstruction (44%), pain in the nasal vestibule (31%), irritative symptoms such as burning nose (26%), and bleeding (17%)^25^. Despite NVSCC should be noticeable at an early stage due to the prominent location in the midface, this disease is often misdiagnosed with inflammatory diseases like vestibulitis or cellulitis, leading to delayed treatment and consequent worsening of survival rates [2, 8].

### Risk factors

Several risk factors have been hypothesised for NVSCC, but the exact role of each one is still under dispute.

Controversial data regarding the role of smoking have been published, ranging from a weak to a strong correlation [8, 30, 31, 34]. In the study by Agger et al., more than half of the patients were smokers or ex-smokers [25]. In a Dutch study on the use of brachytherapy in NVSCC published in 2022, almost three quarters of the population (73,5%) had smoked at least 10 packs/year of cigarettes prior to diagnosis of the disease and nearly one third of patients (32.4%) used to drink at least five units of alcohol per week [8]. In view of its biological behaviour similar to cutaneous carcinomas, also sunlight exposure, especially repetitive sunburn, has been considered to be a risk factor. [8]

Koopmann et al. reported 9 out of 30 patients examined to be teachers, assuming that blackboard chalk could act as a trigger agent, but the correlation has not been proven yet [5].

Studies investigating viral causes of NVSCC are rare. Paulino et al. found no association with Epstein-Barr virus (EBV) [35]. Human Papilloma Viruses (HPV) are established risk factors in the genesis of some head and neck tumours, mostly in the oropharynx [36]. Some authors have supposed this virus may be associated with cancer of nasal cavity (septum) [37] as in several patients SCC were preceded by papillomas. However, evidence is currently too sparse to consider HPV as an etiological factor for NVSCC [25].

### Clinical behaviour

Local patterns of spread of NVSCC are pretty typical and can be understood through the knowledge of anatomy of this region, especially of its cartilages. The most relevant are the septal cartilage, that is single and median, and the alar and the lateral cartilages, paired and lateral, located inferiorly and superiorly, respectively [38]. As known also from laryngeal oncology [39], cartilage itself is resistant to tumor invasion also because it is devoid of blood vessels and fed through the interstitium by direct diffusion from vascularized perichondrium [40]. For these reasons, in NVSCC, especially in early phases, cartilage is not usually disrupted and preferential patterns of spread of these tumors follow cartilagineous surfaces, sliding along them [38, 41]. Moreover, routes of spread also depend on the site of the primary tumor within the NV: cancers of the lateral wall of NV tends to spread sliding through the junction between the alar and lateral cartilages or through the one between lateral cartilages and maxillary and nasal bones, then involving the skin of the ala or of the tip of the nose; primaries of the medial wall of the NV typically spread posteriorly, running along the septal cartilage; tumors of the inferior or medial wall can spread inferiorly to the superior lip or posteriorly, running along the septal cartilage and/or invading the nasal spine and/or the hard palate with bone involvement [38]. As a consequence of that, NVSCC of the lateral wall could be misdiagnosed as skin cancer of the nasal pyramid. To avoid this detrimental mistake, it is imperative to inspect the NV whenever a skin lesion of the nasal pyramid is evident.

Lymphatic drainage occurs mainly via facial, submandibular, and preauricular echelons, less frequently via the submental or digastric nodes [5]. Nodes at higher risk to harbour metastatis are the submandibular ones (level Ib), with several studies reporting pathological nodes exclusively at this site [1, 42].

Data on the incidence of regional metastases (at presentation or during follow up) vary greatly and range from 0 to 40% (Table 1). This variability could be explained by the different inclusion criteria (some studies included also skin cancers of the nasal pyramid) and the different sensitivity of the diagnostic procedures adopted. In the study by Filtenborg et al. published in 2022 on 162 NVSCC, 3,7% of patients had regional metastases at diagnosis and 38% during follow up. In the series published by Czerwinski et al. in 2021, these percentages were 1,8% and 9,8%, respectively [11].

Distant metastases are extremely rare [1]. Filtenborg et al. recorded distant metastasis at diagnosis in only 3 out of 162 patients (1.9%)^7^. However, in most studies the presence of distant metastases is an exclusion criterion, thus hindering a reliable estimate of their occurrence [30].

Distant spread in the further course of the disease is occasionally described [1, 7, 11, 12, 14, 17]. In Czerwinski’s series, 7 distant metastases during the follow up of 225 NVSCC were detected (3.1%); in all cases, they occurred concomitant or subsequent to locoregional recurrence [11].

The organ at higher risk for metastatic spread are the lungs and bones, less frequently the skin and brain [1, 11, 17].

### Classification and staging

There is no consensus on the staging system to use for NVSCC, which limits comparability of published evidence [26]. The three most used systems are listed in Table 2. Nodal staging is based on the American Joint Committee on Cancer/ Union International Cancer Control (AJCC/UICC) N classification [43].

**Table 2:** The three most used staging systems for NVSCC

	Wang [44]	UICC/AJCC - nasal cavity and ethmoid sinus (8th) [43]	AJCC - nonmelanoma skin cancer of H&*N* (8th) [43]
**T1**	The lesion is limited to the nasal vestibule, relatively superficial, involving one or more sites within the vestibule.	Tumour restricted to one subsite of nasal cavity or ethmoid sinus, with or without bony invasion.	Tumor ≤ 2 cm in greatest dimension.
**T2**	The lesion has extended from the nasal vestibule to its adjacent structures, such as the upper nasal septum, upper lip, philtrum, skin of the nose and/or nasolabial fold, but not fixed to the underlying bone.	Tumour involves two subsites in a single site or extends to involve an adjacent site within the nasoethmoidal complex, with or without bony invasion.	Tumor > 2 cm and ≤ 4 cm in greatest dimension.
**T3**	The lesion has become massive with extension to the hard palate, buccogingival sulcus, large portion of the upper lip, upper nasal septum, turbinate and/or adjacent paranasal sinuses, fixed with deep muscle and bone involvement.	Tumour extends to invade the medial wall or floor of the orbit, maxillary sinus, palate, or cribriform plate.	Tumor ≥ 4 cm or minimal erosion of the bone or perineural invasion or deep invasion.
**T4**	-	**T4a**: Tumour invades any of the following: anterior orbital contents, skin of nose or cheek, minimal extension to anterior cranial fossa, pterygoid plates, sphenoid or frontal sinuses. **T4b**: Tumour invades any of the following: orbital apex, dura, brain, middle cranial fossa, cranial nerves other than V2, nasopharynx, or clivus	**T4a**: Tumor with extensive cortical or medullary bone Involvement **T4b**: Tumor with invasion of the base of the cranium or invasion through the foramen of the base of the cranium

AJCC, American Joint Committee on Cancer. UICC, Union International Cancer Control

It is worth mentioning that AJCC/UICC staging system does not recognize a specific classification for NVSCC, which are classified together with those of the rest of the proper nasal cavity [43]. It is a relevant drawback, since NVSCC display peculiar pattern of spread and prognostic factors which could not be properly captured by classifications that have been developed for other tumor sites [41]. For example, skin invasion upstages to cT4a. However, this tumour extension is very frequent in NVSCC also in the early phase of tumour growth (through the nasal valve, mostly deep to alar and superficial to lateral nasal cartilage), and if limited it has not a relevant impact on prognosis, thus not justifying the advanced T class [19]. On the other hand, bony invasion can be present also in cT1 tumours according to the AJCC, while it is a hallmark of locally advanced NVSCC bearing a poorer prognosis [41].

In 1976, Wang proposed a classification of primary lesions, specific for the nose vestibule and focusing on the extension to the proximal structures [44]. Some authors criticised this classification for the absence of tumour size and volume, which were reported to be a significant prognostic factor for regional recurrence [2]. However, it proved to predict prognosis better than other T classifications (Table 1) and currently it is one of the most used, especially in more recent studies [45].

Starting from the consideration that cartilage is not usually disrupted in the early phases of NVSCC, instead guiding the tumor spread, Bussu et al. proposed a new T classification, which follows the anatomical concepts of disease progression (i.e., cartilage and bone invasion), is 4-graded (in accordance with AJCC/UICC T classifications), but still needs to be externally validated (Table 3) [41]. The identification and validation of an efficient, reproducible, and easy-to-apply classification system is an unmet clinical need; in fact, the wide use of such a system would inevitably improve the quality of treatments and favour comparison among different therapeutic strategies.

**Table 3:** Staging system for primary NVSCC proposed by Bussu et al. [41]

	Bussu et al. proposed classification [41]
**T1**	The lesion is limited to the nasal vestibule internal surface (skin and or mucosa).
**T2**	**T2a**: The lesion invades superficial structures outside the nasal cavity (skin and subcutaneous) and in particular upper lip, philtrum, skin of the nose and/or nasolabial fold, but does not destroy cartilage, nor invades bony structures, nor structures beyond the plane of the pyriform aperture (septum, lateral wall, turbinates, etc.) **T2b**: Disruption of cartilages is evident, without invasion of bony structures, nor of structures beyond the plane of the pyriform aperture (septum, lateral wall, turbinates, etc.)
**T3**	The lesion extends beyond the pyriform aperture (septum, lateral wall, turbinates, etc.)
**T4**	**T4a**: The lesion invades bony structures as hard palate, nasal bones, frontal process of the maxilla, ethmoid, and the orbit **T4b**: Tumor invades any of the following: orbital apex, dura, brain, anterior and middle cranial fossa, cranial nerves other than (V2), nasopharynx, or clivus

### Treatment

A gold standard protocol defining the most appropriate treatment of NVSCC is still lacking.

Early-stage tumour, that is T1 (Wang) or T1-T2 (AJCC/UICC), can be treated with single modality, either radiotherapy (RT) or surgery [23, 25, 28].

Regarding advanced stage lesions, there is general consensus that a multimodality approach, consisting of surgical removal and adjuvant treatments, would be the best choice [5, 12, 17, 19, 22, 26]. Post-operative RT on primary site is suggested in case of high-risk histopathological features (inadequate surgical margins, perineural invasion, deep soft tissues and bone infiltration), in patients with regional metastases at the time of diagnosis, and it might be considered for recurrent disease [12, 23].

Primary concurrent chemoradiation (CRT) could also be considered as an organ preserving therapy in selected advanced cases, although evidence is very sparse. In a recent study by Eberle et al. on patients with NVSCC and anterior nasal cavity treated with carbon ion radiotherapy boost (CIRT-B) combined with volumetric intensity modulated arc therapy (VMAT) with photons, patients with advanced stage tumors or nodal involvement received cisplatin chemotherapy, administered simultaneously to photon treatment (40 mg/m2 weekly) [9]. The median organ-preserving survivals at 6 and 24 months were 100% and 83.3%, respectively, for the whole studied group. Despite this, it is necessary to specify that patients who received this treatment were only 8 out of 21. Furthermore, 4 of 21 patients had tumors with different histology from SCC, and tumor histology of the patients that underwent CRT was not specified.

### Surgery and reconstruction techniques

Surgical approach to NVSCC can be external, endoscopic or combined, depending on site, extension of the lesion and planned reconstructive strategy. Introduction of endoscopic-assisted techniques allows magnification of the surgical field and improves delineation of appropriate surgical margins, especially the posterior-septal one [5, 12]. Endoscopic surgery is also useful in reconstruction: septal chondromucosal flaps is often used to reconstruct the inner lining. Endonasal resection has been described for AJCC/UICC T1 or T2 vestibular carcinoma giving good cosmetic appearance and oncological results [24]. Extensive tumour infiltration, however, makes an exclusively endoscopic procedure unfeasible. That is why surgeons should be skilled in endoscopic, external transfacial approaches and in rhinoplasty, to achieve a tumor-customized appropriate resection [12].

In the case of total rhinectomy, many authors state that the optimal reconstruction is a bone-anchored nasal prosthesis, with good patient’s satisfaction [12, 15]. In case of small defects, primary closure can achieve satisfactory results. Defects involving only the skin can be reconstructed using local or regional flaps. If bone or cartilage are involved, reconstruction can be aided by cartilaginous graft (i.e., auricular conca, costal cartilage). Full thickness defects of nasal structure can be repaired with combined local or regional flaps (e.g., nasolabial or nasofrontal flap for the external surface and septal chondromucosal flap for the inner one), with or without grafts. Very large defects may need free flaps [2, 12], but generally cosmetic outcomes are worse than those of a prosthesis.

Timing of eventual reconstruction can vary and can be individualised. Delayed reconstruction is generally recommended to avoid failure due to early tumor recurrence [2, 33, 46]. Samaha et al. suggested a delay of minimum 12 months from the primary treatment [33]. Teichgraeber and Goepfert recommended a 2 years observational period due to aggressive nature of the tumours, with a prosthetic rehabilitation as interim measure [46]. In addition, this strategy could provide the patient with time to evaluate the prosthetic rehabilitation, and some were satisfied and opted against a new surgical procedure for reconstruction [33].

### Radiotherapy

Nonsurgical approaches achieve good oncological results, with 5-year local control rate of 95–98%, and generally better cosmetic outcomes when compared to surgical excision [14, 15, 28, 29, 31, 42, 45]. In the absence of oncological evidence supporting one versus another modality, functional and cosmetics outcomes are paramount in the decision-making. Specifically, reconstruction of the nose tip is notoriously extremely difficult [41]; therefore, in case of tumors that would require the resection of the nasal tip, authors advocate better cosmetic results after irradiation compared to surgery [25, 28, 30, 41, 44].

Considering that nose cartilage itself is generally quite resistant to radiation, many authors advocates RT in the treatment of NVSCC also as primary treatment in early-stage tumours [41]. Both External Beam RadioTherapy (EBRT) and Interventional RadioTherapy (IRT, previously known as brachytherapy) are used; the latter is increasingly employed in clinical practice and several authors support its role as the treatment of choice for early-stage NVSCC (Table 1). IRT generally gives the patient excellent cosmetic outcomes [41].

IRT allows for a higher tumour radiation dose, while limiting exposure of the surrounding tissues through the positioning of catheters directing RT to specific site [47]. It is a multidisciplinary tool that requires cooperation between surgeons and radiation oncologists during every phase, from the recommendation and implantation in the operating theatre, to the prescription and dose painting [41].

In the largest up-to-date multicenter analysis of 225 T1-T2 NVSCC, IRT achieved superior local control compared with EBRT (3-years local control 95% vs. 71%, *p* < 0,01), with a 3-y overall survival of 88%. Patients treated with IRT had a lower risk of salvage total rhinectomy compared to the EBRT group (3-years survival with preserved nose 82% vs. 61%, *p* < 0,01)^11^.

Acute irradiation side effects are exceedingly common in RT of NVSCC and they include dermatitis and desquamation of nasal skin and nasal vestibule, mucositis of nasal and oral cavity, and nasal crusting [31]. The main reported long-term toxicity of IRT in this area is known to be chondronecrosis and consequent septal and even alar perforations [21]. In the series by Czerwinski et al., 5-year RT-induced long-term toxicity was 29%. Radiation ulcers, septal defects and chondronecrosis occurred in 24%, 10% and 4% of cases. Noticeably, up to 50% of all complications was self-limiting or recovered with treatment [11]. Late sequelae can include RT-induced malignancies: Langedjik et al. in their study of 56 patients who underwent primary RT for NVSCC observed 1 case of post-RT sarcoma in NV [30].

Different brachytherapy techniques (intracavitary, interstitial, or a combination, with template or with mold) are described [8]. Chondronecrosis are reported more frequently when an interstitial delivery is chosen, than in endocavitary/mold. This finding suggests that the mechanical damage and interruption of the perichondrium which feeds the cartilage by the implants in the interstitial delivery is relevant, possibly more than the well-known effect of the dose [21, 41]. According to Bussu et al., this complication can be avoided by an anatomic implantation of the plastic tubes which should lie along the subperichondral planes, exploited for the surgical dissection in the rhinoseptoplasty [41]. On the other side, intracavitary catheters could increase radiation on mucosa, leading to a possible increase of mucosal toxicity (i.e., ulcers). In the study published by Scheurleer et al. in 2022 on 67 patients with NVSCC, mold-based combined intracavitary and interstitial brachytherapy technique was employed, limiting the dose on mucosa, without the need for an invasive approach with many interstitial catheters [8]. The oncological outcomes were excellent: 5-year locoregional recurrence-free survival, disease-specific survival, and OS were 91.1%, 96.1%, and 66.2%, respectively [8]. Which technique yields the best oncological outcomes and most beneficial toxicity profile in the treatment of NVSCC is still to be elucidated. A prospective, European multicenter registration study on this topic is soon to be started [8].

As previously reported, in 2022, Eberle et al. published a study that represents the only experience currently available in the literature about the use of radiotherapy with CIRT-B, combined with VMAT, for malignant tumours of the nasal vestibule [9]. Most lesions were Wang T2 or T3 tumours and 17 out of a total of 21 were SCC. 2-year overall survival and local control were 83,3% and 84%, respectively, and the treatment was overall well tolerated. Although it is still a pioneering approach, carbon ions therapy seems to be a good option as an organ-preserving therapy in these tumors [9].

### Elective nodal treatment

In patients with head and neck cancer, cervical nodal metastases are one of the most important prognostic indicators, which indeed in case of positive nodes, decreases by approximately 50%. It is recommended that when the probability of occult cervical lymph node metastasis outweighs 20%, the neck should be electively treated [48].

As previously outlined, lymphatic drainage of the nasal vestibule is primarily through the submandibular, facial, and preauricular nodes. Less commonly, the submental, anterior parotid, or digastric lymph nodes are affected [49]. Scurry et al. published a meta-analysis of nasal cavity SCC with and without prophylactic neck treatment, irrespective of the type of treatment of the primary tumour. They observed a 18.1% rate of regional recurrence, approaching the aforementioned 20% cut-off [50]. However, the studies reviewed included also non-vestibular tumours. In literature specifically regarding NVSCC, the incidence of synchronous or delayed neck nodal metastasis ranges between 3 and 40% (Table 1) [49]. This heterogeneity explains the discrepancies noted between authors recommendations of surveillance, elective nodal dissection or prophylactic radiotherapy for the management of cN0 NVSCC [17, 26]. In any case, this incidence of metastasis warrants thorough meticulous examination of the neck at first diagnosis and during follow-up, especially during the first 2 years from primary treatment, because loco-regional recurrences occur mostly within this period [12, 49]. As outlined by Talmi et al. [49], the series reporting high rates of metastasis at presentation and low incidences in follow-up probably examined their patients well and did not recommend elective neck dissection. Conversely, the series reporting high rates of delayed metastasis and low incidence of N + at presentation raise the question on how the neck has been studied before treatments [20, 25, 32]. So, adequate radiological examination of the neck and fine needle aspiration cytology in case of suspicious nodes may obviate the need for elective neck treatment [49]. In case of clinically positive nodes, a therapeutic neck dissection is the treatment of choice [2].

Lambertoni et al. analysed a series of 45 NVSCC surgically treated from 2010 to 2018 and identified a specific “high-risk” group of patients affected by advanced stage (Wang T2-T3) and aggressive (G2-G3) tumours (20 patients); all neck recurrences were detected in this group [12]. In other studies, tumor diameter > 1.5 cm or volume > 2.3 cm [3] were identified as risk factors for regional failure [11, 21]. In patients with tumors with these characteristics, therefore, elective treatment of the neck should be considered. However, the topic is still debated: regional control rates after salvage treatment are reported to be very high, with a 3-years loco-regional control of 96%^11^. Since elective neck treatment would provide a minor increase in ultimate regional control at the cost of increased morbidity, some authors do not recommend it even in patients with large primary tumour volume [11].

When properly indicated, a prophylactic neck treatment could be achieved either with EBRT or surgical dissection. The former is advisable when RT is planned also on the primary site, the latter is preferable when a surgical violation of the neck is expected, i.e. when a microvascular free-flap reconstruction is planned [49]. If the primary tumour is treated with IRT, neck dissection can be performed in the same session as implantation [41].

The extent of neck treatment in patients with cN0 and cN + tumours is also a debated issue. Concerning surgery, the supraomohyoid neck dissection (level I-III) is adequate unless there is gross disease at level III [49]. It should be performed bilaterally for lesions involving the midline because septo-columellar cancers tend to develop bilateral regional recurrence [12]. Despite the unknown incidence of parotid nodal involvement, prophylactic parotidectomy is excessive and should be performed only in case of gross disease in parotid nodes. [49]

In case of postoperative or elective neck irradiation, generally the entire neck along with the parotid area and retropharyngeal nodes would be included in the radiation field [49].

### Survival and prognostic factors

Reported 5-year OS of NVSCC in literature vary from 50–100%^5^. Survival and locoregional control rates of all studies about this topic published since 2000 to date are shown in Table 1.

Over the years, several factors have proven to impact on the prognosis of this disease. The most important is T classification, especially when classified according to Wang. The classification proposed by Bussu et al. (Table 3) has the potential to improve the staging and could allow to a better prognostic stratification compared to Wang’s and AJCC/UICC ones [41, 51], but still it warrants external validation and further analysis of survival data is needed [51]. 3-year survival rate for T1, T2 and T3 (Wang) was reported to be 83%, 71% and 50%, respectively [5]. In the study by Agger et al., outcomes for T3 Wang tumours were poor with a 5-year disease-specific survival of 39% and an OS of 0%, while these percentages were 83% and 61% for T1 and 63% and 43% for T2, respectively [25]. Also, tumor dimension and volume has been reported as prognostic factors. In the large series of Czerwinski, in univariable analysis, both tumour diameter > 1.5 cm and T2 stage were found to be significant risk factors for regional recurrence, with 3-year regional control rates of 83% and 81%, respectively [11].

When surgery is performed, it is crucial to remove the tumour with adequate margins. Insufficient surgical margins have been reported in several studies to be predictor for a locoregional recurrence [10, 12, 23]. In the series of Lambertoni et al. 5-year recurrence-free survival was 69% in case of adequate resection and dropped to 33,3% in case of positive margins [12].

Regarding tumour extension, infiltration of the deeper tissue layers and, in particular, involvement of the spina nasalis anterior are associated with a poorer prognosis [26]. Tumor arising from septum/columella seems to be more aggressive with worse locoregional control rate in respect to other subsites. This trend could be explained by the difficulty in balancing treatment and cosmetic outcomes in this midline anatomical region and could justify a more aggressive treatment when dealing with this entity [12]. Tumours of this subsite also tend to metastasise bilaterally in the neck [12].

Jeannon et al. showed that nodal status has a strong prognostic predictive value [1]. In their study, a 5-year OS of 40% was described for patients with positive nodal status, while patients without cervical metastases had a 5-year OS of 65%. Horsmans et al. also demonstrated lower OS and recurrence-free survival in patients with positive nodal status (62% vs. 0%; and 93% vs. 0%, respectively) [42]. Lymph node metastases were also classified as prognostically unfavourable in other studies [5].

Most locoregional recurrences occur within 2 years from primary treatment and in all publications only one case of recurrence after 3 years (34 months after primary treatment) has been reported, in Czerwinski’s series of 225 patients [11]. This suggests follow-up duration, at least in patients with early-stage tumours, could be limited to 3–5 years [8].

Koopman et al., in their series, observed that high grade was significantly associated with poorer OS (5-year OS 100% in low-grade tumors vs. 75% in high-grade ones), but had no significant impact on disease-free survival (DFS) [22].

Finally, few molecular prognosticators have been reported so far. Vital et al. found that mutated p53 pattern on histological examination, that was present in half of the patients, is a negative prognosticator [15]. Conversely, positivity for PD-L1 in tumour cells was associated with a favourable DFS [16].

## Conclusions

Nasal vestibule should be considered a distinct site of nose and paranasal sinuses, with specific T staging. A conventional, widespread accepted, user-friendly anatomical definition of this site is paramount to avoid misclassifications and mistreatments.

There are three options available for the treatment of the primary lesion in NV SCC (surgery, EBRT, IRT), without any clear difference in term of oncological outcomes. IRT is increasingly employed due to its promising results in terms of cosmetic and functional outcomes. Multidisciplinar discussion is crucial to define the best patient-tailored therapeutic strategy.

Elective neck treatment might be advocated in the case of high-risk patients, although its exact indications are still debated.

Given the rarity of the disease, further studies are warranted to better define the management of these tumours.
